# Graphene nanosheets decorated with heterostructured ruthenium sulfide as catalyst for enhanced hydrogen evolution reaction

**DOI:** 10.1371/journal.pone.0311885

**Published:** 2024-12-12

**Authors:** Warisha Naseeb, Muhammad Kaleem Khosa, Awal Noor, Sadaf Qayyum, Shaowei Chen

**Affiliations:** 1 Department of Chemistry, Government College University, Faisalabad, Pakistan; 2 Department of Chemistry and Biochemistry, University of California, Santa Cruz, CA, United States of America; 3 Department of Basic Sciences, Preparatory Year Deanship, King Faisal University, Al-Hassa, Saudi Arabia; Minia University, EGYPT

## Abstract

In present findings, a simple pyrolysis technique was applied to decorate S and N doped graphene with RuS_2_-CoO nanoparticles synthesizing a heterostructured nanocomposite RuS_2_-CoO@SNG. XPS results demonstrate the elemental composition of these nanomaterials with the hint of metal-metal charge transfer phenomenon likely due to heterostructure composition. These modifications led to a significant active surface area resulting in elevated electrocatalytic performance. In comparison to benchmark Pt/C at -60 mV in 1 M KOH, hydrogen evolution reaction (HER) reached at current density around 10 mA cm^-2^ at -90 mV overpotential. The stability test displayed excellent results with a decrease of 2 mV in overpotential at current density of 10 mA cm^-2^. Results indicate that such heterostructured nanocomposites can be used as an effective catalyst for HER.

## 1. Introduction

The global energy consumption of human society should, supposedly, be satisfied by non-renewable fossil fuels (coal, gas, oil). In 2022, the fossil fuels accounted for approximately 84% of world energy consumption [1] specifically oil accounting for 31%, natural gas for 24% and coal for 27%. By 2050, the global energy demand will increase by 50%, this inevitable increase in energy demand in past few years is due to increase in population and economic, commercial growth [2]. In addition, this energy crisis is also directly and indirectly related to increase in pollution. This requires a decrease in the production of greenhouse gases e.g., CO_2_. Thus, these emerging challenges for humankind require development of a new, sustainable, clean and environmental-friendly energy resource [3–7]. Hydrogen has appeared as a new clean and sustainable energy resource owing to no greenhouse gas emission during the water electrolysis process [8]. One of the limitations of electrolysis of water across hydrogen evolution reaction (HER) includes low hydrogen production efficiency i.e., high energy consumption and low hydrogen evolution rate. For minimizing the HER overpotential [9] highly efficient catalysts are needed. The catalyst of choice for the electrochemical reactions is noble metal based. For developing noble metal electrocatalyst the major goals are reduced loading of metal and high activity. At present, the benchmark electrocatalyst is Platinum metal. The limitation for platinum-based catalysts is that Pt is expensive and less abundant in nature [10]. Water electrolysis is done in both basic and acidic media but the HER performance of Pt catalyst is lesser in basic media as compared to acidic media due to lower proton concentration in alkaline electrolyte [11]. The initial water dissociation which generates proton in alkaline conditions results in lethargic rate of reaction for HER. Therefore, there is a need for non-Pt, low-cost catalyst which can subdue the energy barrier resulting in an increase in the HER activity kinetics in basic media [12, 13]. Ruthenium is the promising alternative to Pt as its hydrogen adsorption energy is similar to that of platinum and reduced cost half than that of Pt. In addition, ruthenium is adaptable to show stability and can work efficiently in different electrolytes as compared to Pt [14]. Moreover, in comparison to traditional catalysts ruthenium-based catalysts are more environmentally sustainable due to efficiency and recyclability, if lifecycle management practices are taken into account from production to disposal [15].

Ruthenium catalysts often operate at lower temperatures and pressures than other catalysts, thereby reducing energy consumption. In addition, these catalysts can be highly selective, resulting in fewer by-products and reducing the need for energy-intensive purification steps.

Additionally, heteroatom (S, B, P and N) doping of graphene proves to be a successful tactic for improving catalyst performance. The method gets more the active sites and modifies charge density of carbon [16]. These heteroatoms act as the nucleation sites and anchor the metal nanoparticles [17], improving the electrocatalytic activity of the nanomaterial. Due to special layered structure, the heterostructures have stable electrochemical properties and unique electronic orbitals which produces low overpotential during the HER process to help the reaction proceed rapidly. Heterostructures [18] facilitates the interfacial charge transfer and manipulate the valence states enhancing the electrocatalytic performance [19].

Vignesh et al. [20] prepared heterostructured composite of ZnS quantum dots with Co_3_O_4_ Coupling with carbon nitride sheets through facile calcination and hydrothermal technique. The heterojunction between Co_3_O_4_ and ZnS quantum dots on g-C_3_N_4_ leads to a low overpotential of -304 mV at the current density of 10 mA cm^-2^ for hydrogen evolution reaction (HER) in acidic electrolyte. High HER performance was attributed to ample amount of active sites providing high surface area due to synergistic effect of heterostructured ZnS and Co_3_O_4_ growth on g-C_3_N_4_ composite which reduced the interfacial resistance. Li et al. [21] synthesized FeNi flower-like heterostructure on nickel foam substrate for boosting hydrogen evolution reaction (HER) through a simple dip-coating method. The heterostructure between Fe and Ni lead to high HER performance with overpotential to be as low as 228 mV to approach 100 mA cm^-2^ in 1 M KOH and was stable enough to maintain the current density for 10 h. The prominent HER activity was ascribed to the synergistic effect involving Fe and Ni leading to large catalytic surface area.

Zhang et al. [22] synthesized ruthenium and nickel oxide heterostructures by self-templated strategy giving a lowest η_10_ of 14.5 mV. The electrocatalytic activity was attributed to inter-doping and synergistic induction potential due to heterostructure formation of Ru-NiO. Table 1 displays a comparison of these nanocomposites with literature.

**Table 1:** Comparison of electrocatalytic performance with literature.

Catalyst	HER ɳ_10_/V	Media	Reference
Zn-NiCoP hybrid material	0.15	1M KOH	[36]
Ru-NiCo_2_O_4_ nanosheets	0.23	1M KOH	[37]
MoS_2_ nanosheets vertically on the electro-exfoliated blackphosphorus	0.126, 0.237	1M KOH, 0.5M H_2_SO_4_	[38]
Ru@RuO_2_ nanorods	0.137	0.1 M KOH	[39]
Ru-Doped CuO/MoS_2_ Nanostructures	0.198	1M KOH	[40]
Bimetallic polyoxometalate derived Co/WN composite	0.143	0.5M H_2_SO_4_	[41]
This work	0.090, 0.094	1M KOH, 0.5M H_2_SO_4_	

Within this context, we demonstrate the simple pyrolytic fabrication of heterostructured RuS_2_-CoO@SNG nanocomposites. The interactions between RuS_2_ and CoO results in the formation of heterostructure between them The prepared nanocomposites displayed high electrocatalytic performance with η_10_ to be -90 mV and -94 mV in basic and acidic media, respectively.

## 2. Materials and methods

### 2.1. Preparation of graphene nanosheets decorated with RuS_2_-CoO@SNG

A modified Hummers method [23] was used to synthesize graphene oxide (GO). Briefly, conc. H_2_SO_4_ was used to intercalate graphite flakes and KMnO_4_ (as an oxidizing agent) was slowly added in the mixture keeping temperature below 45°C. To end the reaction, 30% H_2_O_2_ was added followed by centrifugation, washing with dil. HCl and water. The brown colored supernatant was stored and left to dry in oven.

Thiourea (120 mg) and GO (60 mg) were used as precursors to synthesize S, N co-doped GO. Both precursors were sonicated for 24 h and were subjected to hydrothermal process for 12 h in an autoclave at 180°C. The precipitates obtained were designated as SNGO. The RuS_2_-CoO@SNG nanocomposite was prepared by using the SNGO dispersion synthesized before, was put in a flask with RuCl_3_.xH_2_O (0.065 mmol) and CoCl_2_.6H_2_O (0.016 mmol). The flask was heated for 4 h at 90°C, centrifuged at 6000 rpm to get precipitates, which were washed, dried in an oven at 60°C for 12 hr. Then calcinated at 700°C at N_2_ atmosphere with flow rate 150 mL min^-1^ for 3 h. The resultant sample was represented by RuS_2_-CoO@SNG. Following the same procedure, three counter samples were prepared using single metal salt donated as RuS_2_@SNG, CoO@SNG and without any metal salt called as SNG.

### 2.2. Characterizations

Tecni G2 200 kV microscope was used to do the transmission electron microscope (TEM) measurements. Energy dispersive spectroscopy mapping (EDS) and Scanning Electron Microscope (SEM) studies were carried on F 200 G2 Talos TEM. Studies using the smartlab Rigaku diffractometer were conducted by using X-ray diffraction (XRD). Using an X-ray K spectrometer, X-ray photoelectron spectroscopy (XPS) spectra were acquired.

### 2.3. Electrochemistry

The electrocatalytic studies were done on an electrochemical workstation CHI710 accompanied by three electrodes, setting glassy carbon, of 0.196 cm^-2^ surface area that works as an electrode, rod of graphite and reference electrode made up of Ag / AgCl). Reference hydrogen electrode (RHE) was functioned as a standard for calibration for reference electrode (Ag/AgCl) and all potentials. To prepare catalyst ink, the sample (2 mg) was sonicated in isopropanol (1 mL) and Nafion (20 μL) for half an hour. 30 μL volume of on the active electrode, catalyst ink was dropped and cast, providing 0.244 mg cm^-2^ mass loading. at the end, the catalyst’s surface was coated with Nafion (6 μL), after drying it, the electrode was immersed in an electrolyte solution. The EIS measurement was done on Gamry.

## 3. Results and discussion

### 3.1. Structural elucidation

**Fig 1:**
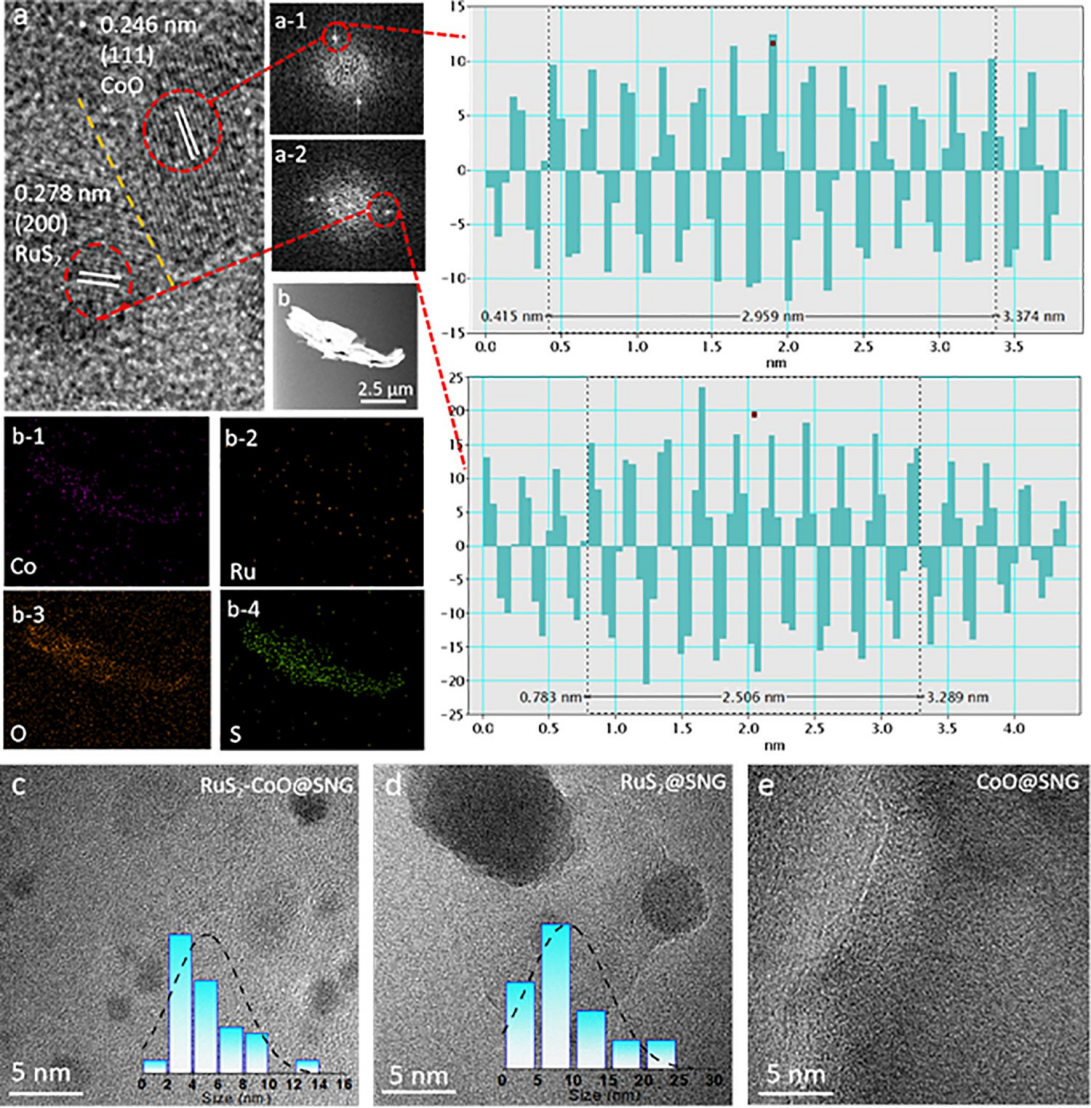
**(a)** High resolution-TEM images of RuS_2_-CoO@SNG presenting lattice fringes of RuS_2_ and CoO. Red circles, **(a-1)** and **(a-2)**, display FFT patterns, respectively, with inter planar distance represented as live profiles of RuS_2_(200) and CoO(111). HAADF-STEM picture of **(b)** RuS_2_-CoO@SNG and EDS elemental mapping of **(b-1)** Co, **(b-2)** Ru, **(b-3)** O and **(b-4)** S. Illustrative TEM representations of **(c)** RuS_2_-CoO@SNG, **(d)** RuS_2_@SNG, and **(e)** CoO@SNG. The histograms are shown as insets in panels **(c)** and **(d)**.

Experimentally, modified Hummers method was used to prepare graphene oxide and then doped with sulfur and nitrogen heteroatoms via hydrothermal reaction at 180°C for 12h. The stacked structure of RuS_2_-CoO@SNG was obtained through refluxing at 90°C with Ru^3+^ and Co^2+^ salts during which hydrolysis of both metal ions occurred resulting in RuS_2_ and CoO species, respectively. Pyrolysis of synthesized product under 700°C and N_2_ atmosphere synthesized RuS_2_-CoO@SNG heterostructure in the main sample. TEM technique was used to characterize the synthesized sample. The high-resolution TEM (HRTEM) measurements (Fig 1) demonstrate visibly different lattice fringes with the existence of interface, presenting interplanar spacings of 0.278 nm which represent cubic RuS_2_(200) (JCPDS card no. 19–1107) [24] and 0.246 nm for cubic CoO (JCPDS card no. 78–0431) [25]. This is also validated in corresponding live profiles. FFT images in Fig 1A manifest the two crystalline domains which are in close vicinity to each other in such a manner that they produce RuS_2_-CoO heterostructures. Fig 1B, the elemental mapping based on EDS, showed the circulation of Co and Ru on graphene nanosheets decorated with O and S elements. The TEM image (Fig 1C) shows many nanoparticles of different sizes scattered on the scaffold between 2 and 8 nm, with a usual size of 5.0 ± 0.8 nm (Fig 1C inset). The TEM image of RuS_2_@SNG (Fig 1D) displays different nanoparticles with larger size in the range of 9 ± 0.5 nm is the mean size, ranging from 5 to 15 nm.

### 3.2. XRD measurements

**Fig 2:**
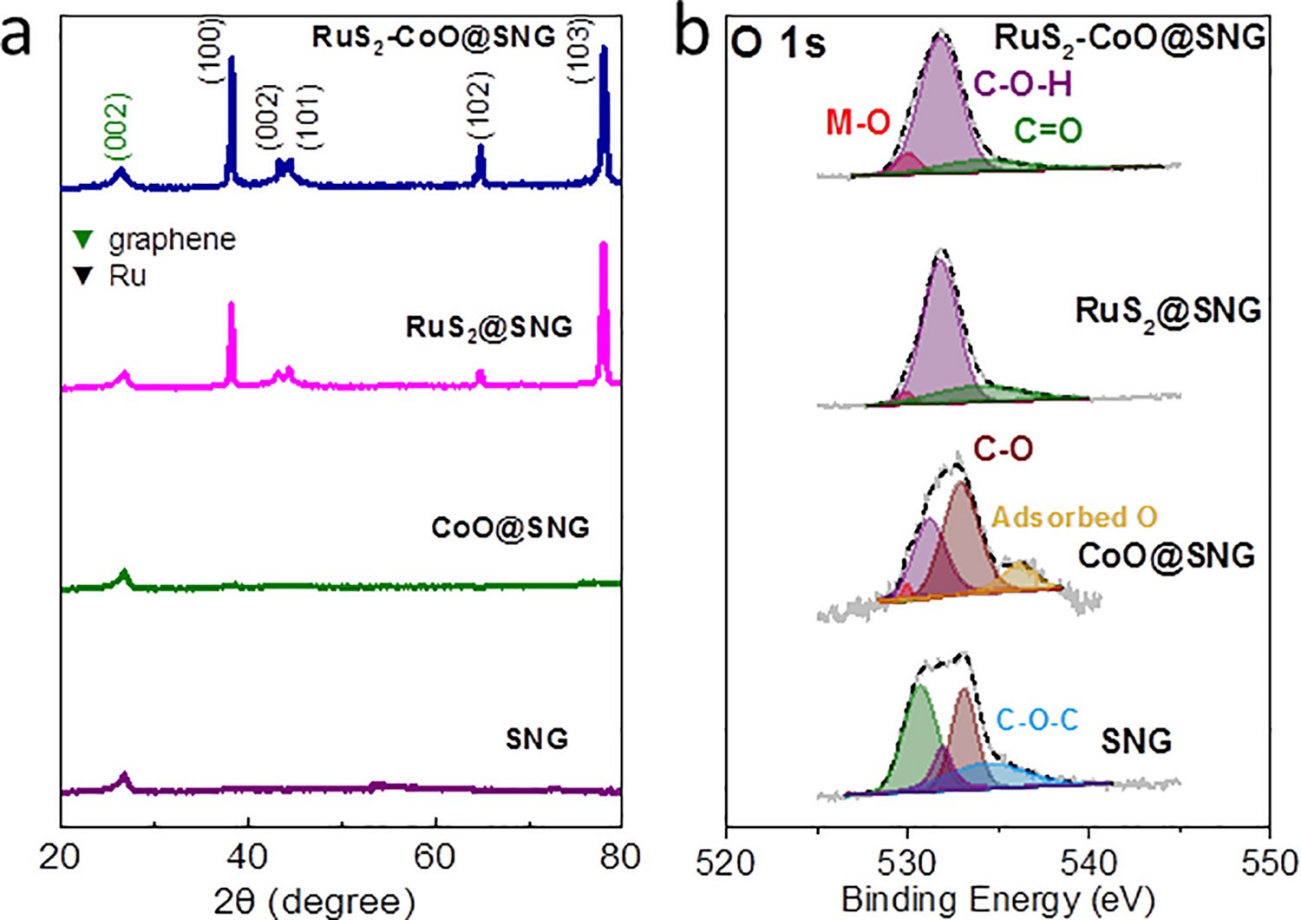
**(a)** XRD Pattern **(b)** PS spectra of O 1s in RuS_2_-CoO@SNG, RuS_2_@SNG, CoO@SNG and SNG. Deconvolution fits are represented by the colored curves and raw experimental data is represented by gray solid curves.

(Fig 2) displays the XRD measurements. Different peaks can be seen in the XRD graphs, with a peak at 2θ = 26.5° present in all four of the graphs, designated to graphene (002) facet (JCPDS card no. 65–6212), implying that Through the technique of pyrolysis, graphene oxide was successfully converted to graphene. The additional peaks can be seen in RuS_2_-CoO@SNG and RuS_2_@SNG XRD graphs with the peaks of hexagonal Ru. No noticeable peak could be found for RuS_2_, suggesting monolayer thickness of RuS_2_ nanoparticles. For CoO@SNG XRD graph, no prominent peak was observed verifying the presence of amorphous structure for the nanocomposite confirming the TEM data results.

### 3.3. XPS studies

**Fig 3:**
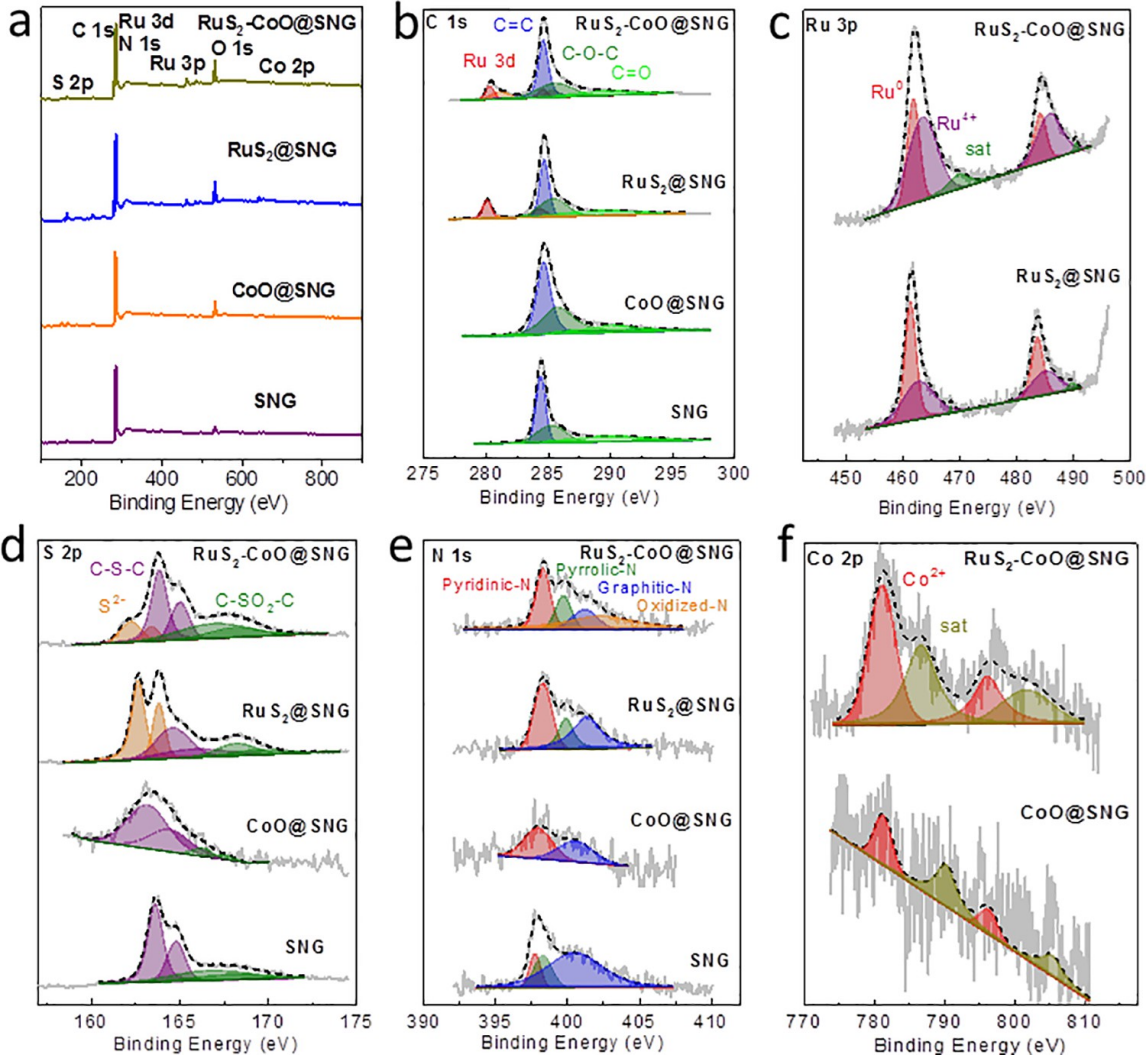
**(a)** XPS survey spectra of all four samples. XPS profiles of **(b)** C 1s **(c)** Ru 3p **(d)** S 2p **(e)** N 1s **(f)** Co 2p electrons of four nanocomposites. Deconvolution fits are represented by the colored curves and raw experimental data is represented by gray solid curves.

XPS studies were done to determine the elemental valence state and their composition. (Fig 3A) displays the survey spectra of the nanocomposites. The significant peaks can be seen at 283 eV (C 1s), 532 eV (O 1s), 398 eV (N 1s) and 165 eV (S 2p). Ru (3d) peak overlapped with C1s peak. The Ru 3p peak can be seen at 461 eV in RuS_2_-CoO@SNG and RuS_2_@SNG nanocomposites spectra while this peak is absent in CoO@SNG nanocomposites spectra. The elemental composition of RuS_2_-CoO@SNG on the basis of integrated peak areas, was estimated to be 80.59 @ C, 1.43%, Ru, 0.37%, Co, 2.26%, N, 12.46%, O and S, 2.86% respectively. Deconvolution of all the four samples give three peaks with sp^2^-hybridization, 284.61 eV for (C = C), 285.55 eV for C-C and 289.33 eV for C = O [26]. The extra peaks were spotted in RuS_2_-CoO@SNG XPS spectra at 280.33/284.53, 281.10/285.30 eV and in RuS_2_@SNG XPS spectra at 280.09/284.29, 280.97/285.17 eV (Fig 3C) [27]. These doublets represent the electrons of 3d_5/2_ / 3d_3/2_ for Ru^0^ and Ru^4+^ species, respectively. These peaks were consistent with the XPS spectra of Ru 3p containing two doublets at 461.91/484.11 and 463.52/485.72 eV for RuS_2_-CoO@SNG; 461.42/483.62 and 462.90/485.10 eV for RuS_2_@SNG [28]. It should be mentioned that the binding energy of Ru^4+^ is higher in RuS_2_-CoO@SNG. In Ru 3d, +0.1 eV, and in Ru 3p, +0.5 eV as compared to Ru^4+^ peaks in RuS_2_@SNG. This shift in binding energy suggests that there is an electron deficit in Ru with RuS_2_-CoO@SNG heterostructure in comparison to RuS_2_@SNG without heterointerface. Co 2p spectra is demonstrated in Fig 2D, where a doublet can be observed for RuS_2_-CoO@SNG at 781.20/796.19 eV and 781.31/796.31 eV for CoO@SNG referable to 2p_3/2_ / 2p_1/2_ Co^2+^ electrons, respectively [29]. As observed, binding energy of Co^2+^ for RuS_2_-CoO @SNG is -0.1 eV lower than that of CoO@SNG. These shifts in the binding energy (Table 2) indicate that electron transfer occurs between Ru and Co in RuS_2_-CoO@SNG. These shifts in peaks implies redistributed charge density due to intimate contact between RuS_2_ and CoO leading to move electrons from Co to Ru in RuS_2_-CoO@SNG due to formation of heterointerface between these two components in contrast to RuS_2_@SNG and CoO@SNG. The idea is to adjust electron density of Ru, resulting in favorable adsorption/desorption energies of intermediates on the catalytic sites which is necessary for high HER performance. Thus, all these helpful modifications like addition of a carbon substrate, heteroatom doping, addition of a second metal is to manipulate the electronic properties of this heterostructure to gain a good HER catalyst.

**Table 2:** Binding energies (eV) and content (%) of Ru and Co species of series by XPS.

Sample	Ru (0)	Ru (4+)	Co (2+)
	280.33 eV	281.10 eV	781.20 eV
RuS_2_-CoO@SNG	0.72 at. %	0.84 at. %	0.204 at%
	461.91 eV	463.52 eV	
	0.46 at%	0.85 at %	
	280.09 eV	280.97 eV	-
	1.09 at. %	0.09 at. %	
RuS_2_@SNG	461.42 eV	462.90 eV	
	0.48 at%	0.40 at%	
**CoO@SNG**			781.31 eV
			0.08 at%

XPS spectra of S 2p (Fig 3D) for all materials, where, peak at 162.22 eV reveals the presence of Ru-S in RuS_2_-CoO@SNG as compared to RuS_2_@SNG where 162.63 eV peak is ascribed as Ru-S peak [30, 31]. CoO@SNG does not display any sulfide peak suggesting no formation of sulfide in the structure. The doping of sulphur in the RuS_2_- CoO@SNG can be identified by 163.38 eV (C-S-C) peak [32]. Pyridine N can be found at 398.28 eV, pyrrole N at 399.68 eV and graphitic N at 401.17 eV (Fig 3E) [33]. Results confirmed the doping of nitrogen into carbon framework successfully [34].

### 3.4. Electrocatalytic study

**Fig 4:**
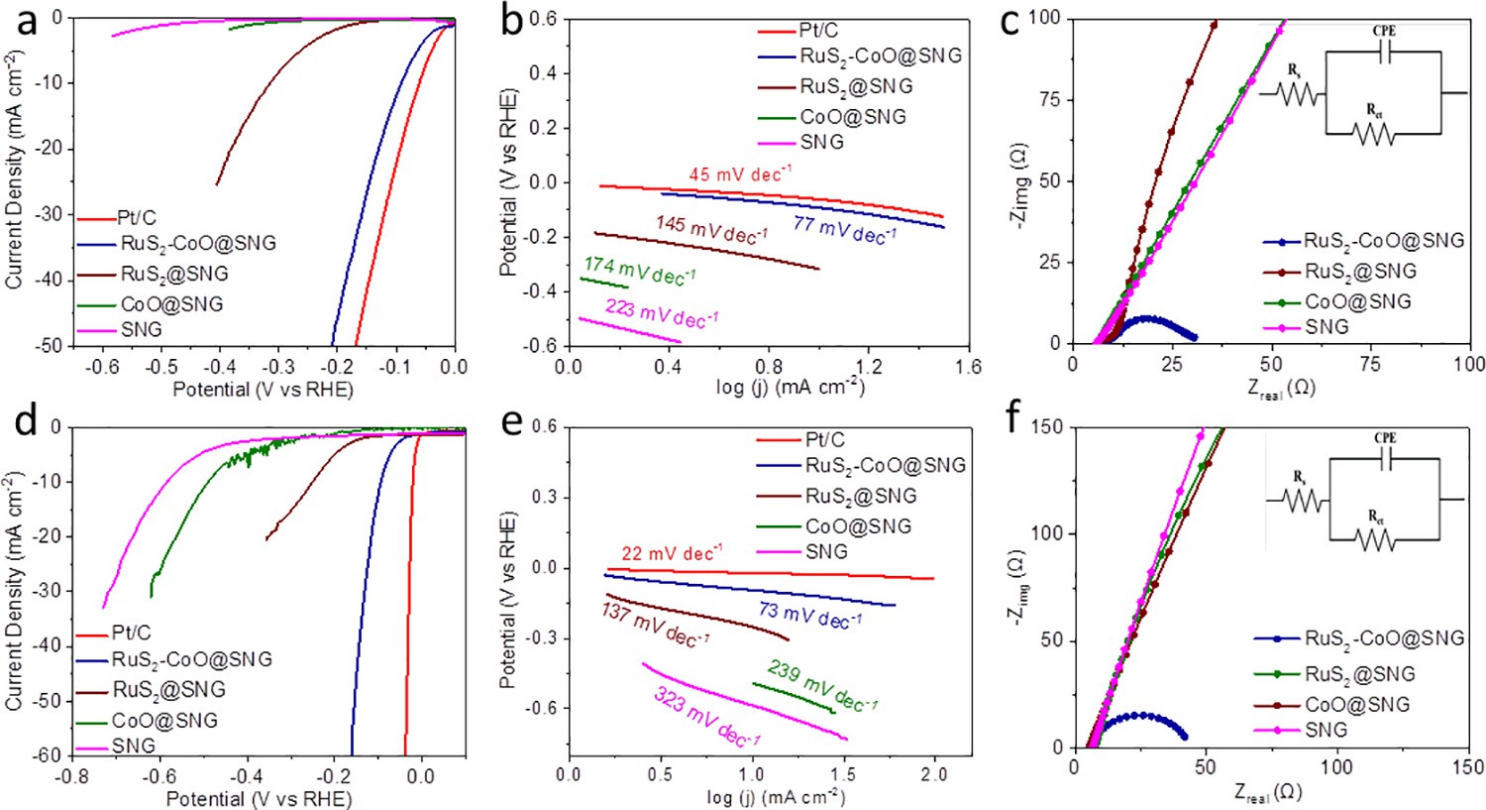
**(a)** Linear sweep voltammetry(LSV) profiles (1 M KOH) @ 10 mV s^-1^ potential sweep rate, 85% iR compensation and 1600 rpm, rate of rotation of **(b)** Respective Tafel plots of all samples. **(c)** The Nyquist plots (1M KOH) at -87 mV overpotential **(d)** LSV profiles (0.5 M H_2_SO_4_) at 10 mV s^-1^ potential sweep rate, 1600 rpm rate of rotation and 85% IR compensation. **(e)** Corresponding Tafel slopes **(f)** Nyquist plots (0.5 M H_2_SO_4_) at -100 mV overpotential. Insets to **(c)** and **(f)** are equivalent circuits showing charge transfer resistance (R_ct_), constant phase element (CPE) and serial resistance (R_S_).

The synthesized RuS_2_-CoO@SNG sample was tested for electrocatalytic HER in N_2_ saturated KOH (1M) as basic and H_2_SO_4_ (0.5M) as acidic media respectively. The polarization curves are presented in (Fig 4A), where η_10_ for RuS_2_-CoO@SNG was found to be just -90 mV in comparison to other three counter samples which displayed η_10_ at -314 mV for RuS_2_@SNG while other two were unable to reach η_10_ value even at small potentials of -0.4 and -0.6 V. This highlights the contribution of RuS_2_-CoO heterojunction on the high performance of nanocomposite with minimum influence of RuS_2_ alone and SNG framework. (Fig 4B) illustrates the corresponding Tafel slopes of polarization curves, the slope for RuS_2_-CoO@SNG was anticipated to be 77 mV dec^-1^, suggesting Volmer-Hevrosky [35] reaction kinetics of HER, in comparison to that of 127 mV dec^-1^ for RuS_2_@SNG and 174 mVdec^-1^ for CoO@SNG. In basic media, the commercial Pt/C exhibited η_10_ at -60 mV and Tafel slope at 45 mV dec^-1^ representing the high performance of heterostructured RuS_2_-CoO@SNG owing to the low difference between HER performance of nanocomposite and Pt/C (Fig 4C). Remarkably, the RuS_2_-CoO@SNG demonstrated high performance in acid electrolyte (0.5 M H_2_SO_4_), displaying η_10_ value to be -94 mV (Fig 4D) while RuS_2_@SNG display η_10_ at -252 mV, CoO@SNG at -495 mV and SNG at -585 mV [36]. The HER reaction kinetics of nanocomposites was estimated by corresponding Tafel plots of polarization curves [37] in acidic media (Fig 4E). The RuS_2_-CoO@SNG nanocomposite exhibited a less Tafel slope (73 mV dec^-1^) demonstrating the suitable HER kinetics in comparison to commercial Pt/C which presented η_10_ to be -60 mV with Tafel slope (45 mV dec^-1^). Nyquist plots (Fig 4F) shows lowest R_ct_ as 37.74 Ω for RuS_2_-CoO@SNG in contrast to RuS_2_@SNG (154.97 Ω), CoO@SNG (5065 Ω) and SNG (6524 Ω) [38].

### 3.5. Stability tests

**Fig 5:**
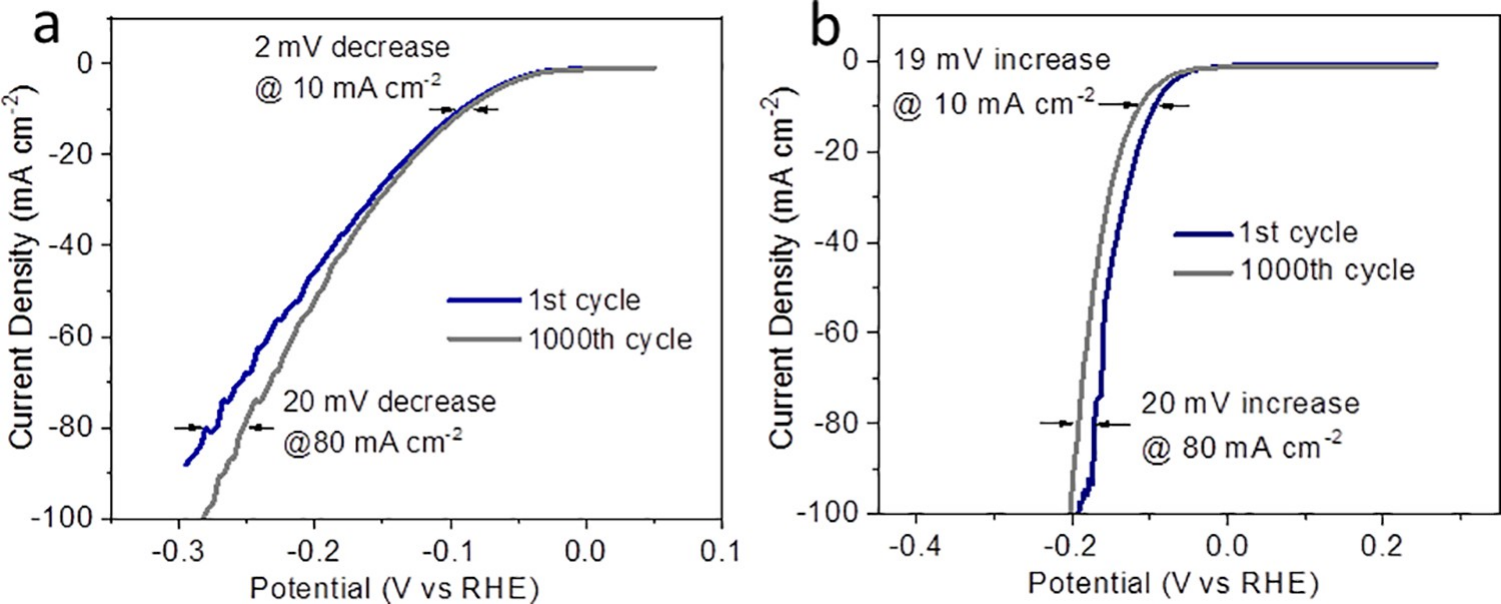
Stability tests of RuS_2_-CoO@SNG in **(a)** 1M KOH **(b)** 0.5 M H_2_SO_4_.

**Fig 6:**
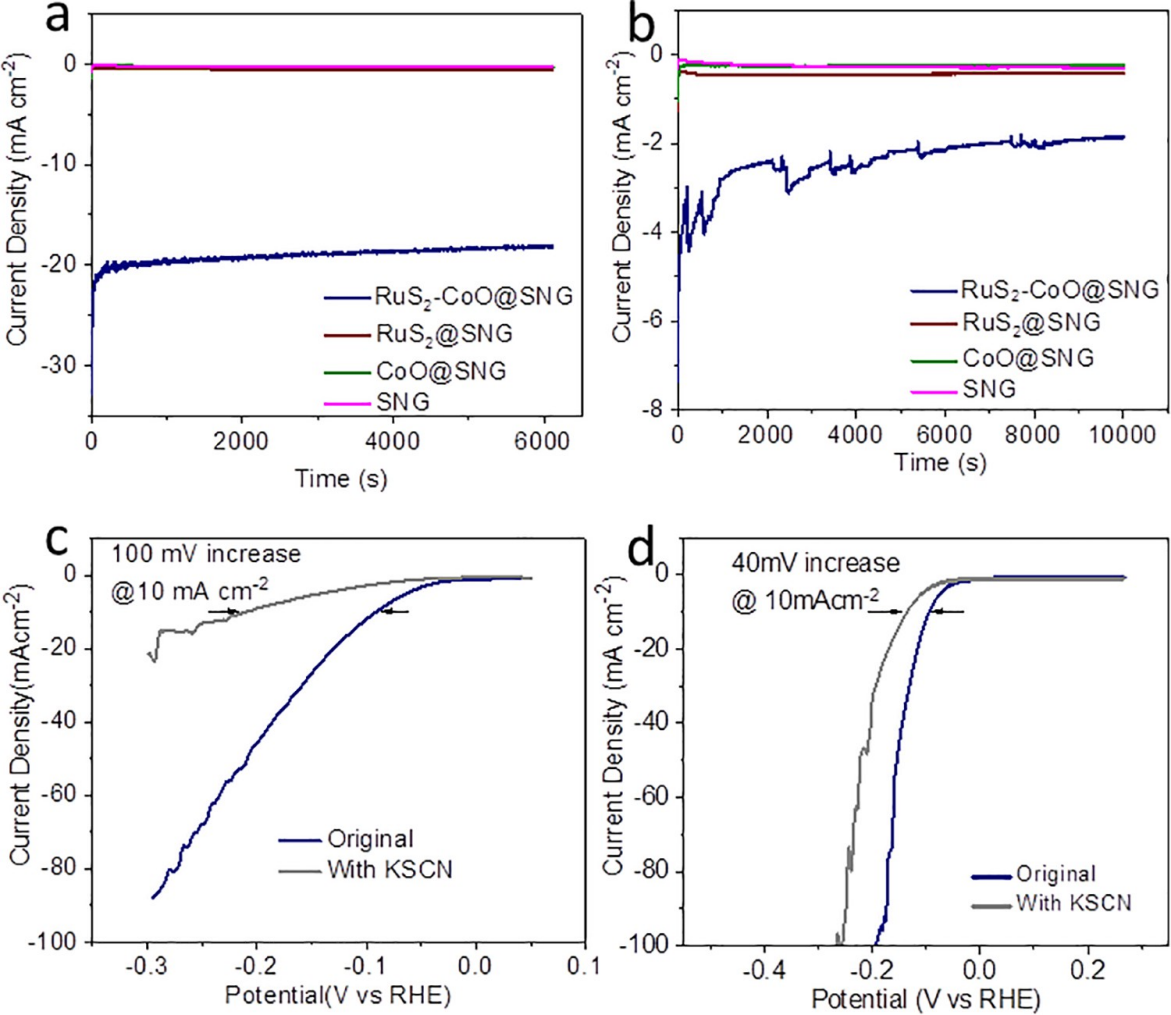
i-t curve of RuS_2_-CoO@SNG in (a) 1M KOH at 153 mV (b) 0.5 M H_2_SO_4_ at 95 mV. KSCN poisoning test of RuS_2_-CoO@SNG in (c) 1 M KOH and (d) 0.5 M H_2_SO_4_.

Stability tests were also investigated in both alkaline and acidic media. After 1000 cycles (1 M KOH), RuS_2_-CoO@SNG nanocomposites displayed remarkable stability with a slight increase in HER performance giving a decrease of 2 mV in η_10_ value (Fig 5A). On the contrary, after 1000 cycles (0.5 M H_2_SO_4_), HER LSV profile of RuS_2_@SNG was decreased giving only 19 mV increase in η_10_ value [39] and 20 mV increase in η_80_ value (Fig 5B). These results suggest that nanocomposites were activated for HER. The cyclic voltammograms were recorded to determine the active surface area within the non-Faradaic region in range of 10–60 mV/s to quantify double layer capacitance (Cdl) [40]. The Cdl value for RuS_2_-CoO@SNG in basic media (1 M KOH) was determined 3.34 mF cm^-2^ as compared to 2.74 mF cm^-2^ for RuS_2_@SNG, 0.025 mF cm^-2^ for CoO@SNG and 0.025 mF cm^-2^ for SNG. Similarly, based on the cyclic voltammograms RuS_2_-CoO@SNG nanocomposites displayed largest ECSA in 0.5 M H_2_SO_4_ with Cdl value to be 8.4 mF cm^-2^ in comparison to 2.3 mF cm^-2^ for RuS_2_@SNG, 0.076 mF cm^-2^ (CoO@SNG) and 0.037 mF cm^-2^ (SNG). The high Cdl value for RuS_2_-CoO@SNG heterostructured nanocomposites in both basic and acidic media, respectively, suggests that it has largest active surface area that resulted in high HER performance due to ease in availability of catalytic active sites [41]. The chronoamperometric tests (Fig 6) displayed that RuS_2_-CoO@SNG nanocomposites preserved 21 mA cm^-2^ current density in alkaline electrolyte at -153 mV overpotential for approximately 2h and 4.7 mA cm^-2^ current density in acidic media for 3h, in contrast to the other three samples which displayed negligible stability.

To identify the location of active sites, poisoning tests (Fig 6) were performed using potassium thiocyanate (KSCN), resulting in decrease of HER activity causing an increase in η_10_ value (1 M KOH) by 100 mV and an increase in η_10_ value (0.5 M H_2_SO_4_) by only 40 mV, implying blockage of heterojunction active area of nanocomposite by SCN^-^ species thus reducing HER activity.

## 4. Conclusions

In this research, heterostructured nanoparticles of RuS_2_-CoO decorated on co-doped graphene scaffold has been prepared through hydrothermal-pyrolysis method. The obtained RuS_2_-CoO@SNG nanocomposite showed high HER electrocatalytic activity in 1 M KOH (η_10_ = -90 mV), a Tafel slope of 77 mV dec^-1^ and η_10_ of -94 Mv in 0.5 M H_2_SO_4_ with a Tafel slope of 73 mV dec^-1^. High HER activity was due to interactions between RuS_2_ and CoO nanoparticles resulting in a heterostructure due to electron transfer from RuS_2_ to CoO. Moreover, doping of graphene with heteroatoms (S, N) cause the dispersion of metal nanoparticles resulting in facile reaction kinetics due to high amount of catalytic active sites. This series of studies highlights the importance of different heterostructured nanoparticles with a specific scaffold (SNG) in developing high performance electrocatalysts in both basic and alkaline conditions for electrochemical energy technologies.

## Supporting information

S1 File (DOCX)

## References

[1] Holechek J. L., Geli H. M. E., Sawalhah M. N., Valdez R., A Global Assessment: Can Renewable Energy Replace Fossil Fuels by 2050? *Sustainability* 14, 4792 (2022). doi: 10.3390/su14084792

[2] Inocencio C. V., Holade Y., Morais C., Kokoh K. B., Napporn T. W., Electrochemical hydrogen generation technology: Challenges in electrodes materials for a sustainable energy. *Electrochem*. *Sci*. *Adv*. 3, 1–52 (2022). doi: 10.1002/elsa.202100206

[3] Pu Z. et al., Single-atom catalysts for electrochemical hydrogen evolution reaction: recent advances and future perspectives. *Nano-Micro Lett*. 12, 1–29 (2020). doi: 10.1007/s40820-019-0349-y 34138058 PMC7770676

[4] Zheng X. et al., Non-carbon-supported single-atom site catalysts for electrocatalysis. *Energy Environ*. *Sci*. 14, (2021). doi: 10.1039/D1EE00248A

[5] Usman M. et al., In Situ Synthesis of a Polyaniline/ Fe-Ni Codoped Co_3_O_4_ Composite for the Electrode Material of Supercapacitors with Improved Cyclic Stability. *ACS Omega* 6, 1190–1196 (2021). doi: 10.1021/acsomega.0c04306 33490777 PMC7818300

[6] Adnan M. et al., Study of magnetic and dielectric properties of ZnFe_2_O_4_/CoCr_2_O_4_ nanocomposites produced using sol-gel and hydrothermal processes. *J*. *Alloys Compd*. 865, 158953 (2021). doi: 10.1016/j.jallcom.2021.158953

[7] Arshad N. et al., Nanoengineering of NiO/MnO_2_/GO Ternary Composite for Use in High-Energy Storage Asymmetric Supercapacitor and Oxygen Evolution Reaction (OER). *Nanomaterials* 13, 99 (2023). doi: 10.3390/nano13010099 36616009 PMC9823737

[8] Zheng Y., Jiao Y., Qiao S. Z., Engineering of carbon-based electrocatalysts for emerging energy Conversion: from fundamentality to functionality. *Adv*. *Mater*. 27, 5372–5378 (2015). doi: 10.1002/adma.201500821 26174510

[9] Suen N. T., Hung S. F., Quan Q., Zhang N., Xu Y. J., Chen H. M., Electrocatalysis for the oxygen evolution reaction: recent development and future perspectives. *Chem*. *Soc*. *Rev*. 46, 337–365 (2017). doi: 10.1039/c6cs00328a 28083578

[10] Lai W. H. et al., General π‐electron‐assisted strategy for Ir, Pt, Ru, Pd, Fe, Ni single‐atom electrocatalysts with bifunctional active sites for highly efficient water splitting. *Angew*. *Chem*., *Int*. *Ed*. 58, 11868–11873 (2019). doi: 10.1002/anie.201904614 31173428

[11] Dinh C. T. et al., Multi-site electrocatalysts for hydrogen evolution in neutral media by destabilization of water molecules. *Nat*. *Energy*, 4, 107–114 (2019). doi: 10.1038/s41560-018-0296-8

[12] Coskun H. et al., Metal-Free Hydrogen-Bonded Polymers Mimic Noble Metal Electrocatalysts. *Adv*. *Mater*. 32, 1902177–1902185 (2020). doi: 10.1002/adma.201902177 32419235

[13] Dai M., Zhao D., Liu H., Tong Y., Hu P., Wu X., Nanostructure and doping engineering of ZnCoP for high performance electrolysis of water. *Mater*. *Today Energy*, 16, 100412–100428 (2020). doi: 10.1016/j.mtener.2020.100412

[14] Bae S. Y., Mahmood J., Jeon I. Y., Baek J. B., Recent advances in ruthenium-based electrocatalysts for the hydrogen evolution reaction. *Nanoscale Horiz*. 5, 43–56 (2020). doi: 10.1039/C9NH00485H

[15] Mori M., Stropnik R., Sekavcnik M., Lotric A., Criticality and Life-Cycle Assessment of Materials Used in Fuel-Cell and Hydrogen Technologies. *Sustainability* 13, 3565 (2021). doi: 10.3390/su13063565

[16] Su J., Yang Y., Xia G., Chen J., Jiang P., Chen Q., Ruthenium-Cobalt nanoalloys encapsulated in nitrogen-doped graphene as active electrocatalysts for producing hydrogen in alkaline media. *Nat*. *Commun*. 8, 14969–14978 (2017). doi: 10.1038/ncomms14969 28440269 PMC5413983

[17] Song H., Wu M., Tang Z., Tse J. S., Yang B., Lu S., Single atom ruthenium-doped CoP/CDs nanosheets via splicing of carbon-dots for robust hydrogen production. *Angew*. *Chem*. *Int*. *Ed*. 60, 7234–7244 (2021). doi: 10.1002/anie.202017102 33438321

[18] Gao X. et al., Ru/RuO_2_ Nanoparticle composites with N-doped reduced graphene oxide as electrocatalysts for hydrogen and oxygen evolution. *ACS Appl*. *Nano Mater*. 3, 12269–12277 (2020). doi: 10.1021/acsanm.0c02739

[19] Liu T., Li A., Wang C., Zhou W., Liu S., Guo L., Interfacial electron transfer of Ni_2_P–NiP_2_ polymorphs inducing enhanced electrochemical properties. *Adv*. *Mater*, 30, 1803590–1803602 (2018). doi: 10.1002/adma.201803590 30285280

[20] Vignesh S., Kim H., Rational Construction of efficient ZnS quantum dots-supported g-C_3_N_4_ with Co_3_O_4_ heterostructure Composite for bifunctional electrocatalytic hydrogen evolution reaction and environmental pollutant degradation. *J*. *Alloys Compd*. 942, 169077 (2023). doi: 10.1016/j.jallcom.2023.169077

[21] Li H., Wang Z., Wang X., Wang T., Synthesis of FeNi-based heterostructure on a nickel foam at room temperature for efficient catalyzing hydrogen evolution reaction. *Mater*. *Lett*. 357, 135757 (2024). doi: 10.1016/j.matlet.2023.135757

[22] Zhang H. et al., Inter-doped ruthenium–nickel oxide heterostructure nanosheets with dual active centers for electrochemical-/solar-driven overall water splitting. *Appl*. *Catal*., *B*. 298, 120611–120622 (2021). doi: 10.1016/j.apcatb.2021.120611

[23] He T. et al., Nanocomposites Based on Ruthenium Nanoparticles Supported on Cobalt and Nitrogen-Codoped Graphene Nanosheets as Bifunctional Catalysts for Electrochemical Water Splitting. *ACS Appl*. *Mater*. *Interfaces* 11, 46912–46919 (2019). doi: 10.1021/acsami.9b17056 31755691

[24] Xu Y., Gao X., Zhang J., Gao D., Nitrogen-doped RuS_2_ nanoparticles containing in situ reduced Ru as an efficient electrocatalyst for hydrogen evolution. *RSC Adv*. 10, 17862–17868 (2020). doi: 10.1039/D0RA02530E 35515613 PMC9053597

[25] Wang L. H., Teng X. L., Qin Y. F., Li Q., High electrochemical performance and structural stability of CoO nanosheets/CoO film as self-supported anodes for lithium-ion batteries. *Ceram*. *Int*. 47, 5739–5746 (2021). doi: 10.1016/j.ceramint.2020.10.160

[26] Alenezi G. T., Rajendran N., Nazeer A. A., Makhseed S., Development of Uniform Porous Carbons From Polycarbazole Phthalonitriles as Durable CO_2_ Adsorbent and Supercapacitor Electrodes. *Front*. *Chem*. 10, 879815–879832 (2022). doi: 10.3389/fchem.2022.879815 35548674 PMC9081769

[27] Medina G. E. B. et al., Hybrid material by anchoring a ruthenium (ii) imine complex to SiO_2_: preparation, characterization and DFT studies. *RSC Adv*. 11, 6221–6233 (2021). doi: 10.1039/D0RA09282G 35423152 PMC8694867

[28] Choi W. et al., Ru-Doped Co_3_O_4_ Nanoparticles as Efficient and Stable Electrocatalysts for the Chlorine Evolution Reaction. *ACS Omega*, 8, 35034–305043 (2023). doi: 10.1021/acsomega.3c04525 37779938 PMC10536866

[29] Kalasina S. et al., Cobalt oxysulphide/hydroxide nanosheets with dual properties based on electrochromism and a charge storage mechanism. *RSC Adv*. 10, 14154–14160 (2020). doi: 10.1039/d0ra01714k 35498444 PMC9051897

[30] Kumar S. et al., Correlating the Influence of Disulfides in Monolayers across Photoelectron Spectroscopy Wettability and Tunneling Charge-Transport. *J*. *Am*. *Chem*. *Soc*. 142, 15075–15083 (2020). doi: 10.1021/jacs.0c06508 32786759 PMC7472521

[31] Dhingra A. et al., Effect of Au/HfS_3_ interfacial interactions on properties of HfS_3_-based devices. *Phys*. *Chem*. 24, 14016–14021 (2022). doi: 10.1039/D2CP01254E 35638717

[32] Di Z. et al., Heteroatom doping effect of Pt/rGO catalysts for formaldehyde abatement at ambient temperature. *Chem*. *Phys*. *Impact*, 5, 100103–100112 (2022). doi: 10.1016/j.chphi.2022.100103

[33] Yan F. et al., Copper coordinated with nitrogen in electrospun carbon nanofibers as a high-performance electrocatalyst for ORR. *Electrochem*. *Commun*. 136, 107245 (2022). doi: 10.1016/j.elecom.2022.107245

[34] Chae G. S., Youn D. H., Lee J. S., Nanostructured Iron Sulfide/N, S Dual-Doped Carbon Nanotube-Graphene Composites as Efficient Electrocatalysts for Oxygen Reduction Reaction. *Materials*, 14, 2146–2158 (2021). doi: 10.3390/ma14092146 33922588 PMC8122905

[35] Bao F. et al., Understanding the Hydrogen Evolution Reaction Kinetics of Electrodeposited Nickel-Molybdenum in Acidic, Near-Neutral, and Alkaline Conditions. *ChemElectroChem*, 8, 195–208 (2021). doi: 10.1002/celc.202001436

[36] Wang Q., Wang C., Du X., Zhang X., Controlled synthesis of M (M = Cr, Cu, Zn and Fe)-NiCoP hybrid materials as environmentally friendly catalyst for seawater splitting. *J Alloys Comp* 966, 171516 (2023). doi: 10.1016/j.jallcom.2023.171516

[37] Yang R. et al., Ruthenium-modified porous NiCo2O4 nanosheets boost overall water splitting in alkaline solution. *Chin*. *Chem*. *Lett*. 33, 4930–4935 (2022). doi: 10.1016/j.cclet.2021.12.058

[38] Liang T., Liu Y., Cheng Y., Ma F., Dai Z., Scalable Synthesis of a MoS2/Black Phosphorus Heterostructure for pH‐Universal Hydrogen Evolution Catalysis. *ChemCatChem* 12, 2840–2848 (2020). doi: 10.1002/cctc.202000139

[39] Jiang R., Tran D. T., Li J., Chu D., Ru@RuO_2_ Core-Shell Nanorods: A Highly Active and Stable Bifunctional Catalyst for Oxygen Evolution and Hydrogen Evolution Reactions. *Energy Environ*. *Mater*. 2, 201–208 (2019). doi: 10.1002/eem2.12031

[40] Maiti A., Srivastava S. K., Ru-Doped CuO/MoS_2_ Nanostructures as Bifunctional Water-Splitting Electrocatalysts in Alkaline Media. *ACS Appl*. *Nano Mater*. 4, 7675–7685 (2021). doi: 10.1021/acsanm.1c00791

[41] Men L. et al., Bimetallic polyoxometalate derived Co/WN composite as electrocatalyst for high-efficiency hydrogen evolution. *Int*. *J*. *Hydrogen Energy* 47, 27452–27459 (2022). doi: 10.1016/j.ijhydene.2022.06.061

