# Supplementary material for: Graphene nanosheets decorated with heterostructured ruthenium sulfide as catalyst for enhanced hydrogen evolution reaction
Source: PLoS One. 2024 Dec 12;19(12):e0311885. doi: 10.1371/journal.pone.0311885 (PMC11637258; doi:10.1371/journal.pone.0311885)
Supplement: S1 File — (DOCX) [file pone.0311885.s001.docx]

**Supporting Informations**

**Graphene nanosheets decorated with heterostructured ruthenium sulfide as catalyst for enhanced hydrogen evolution reaction**

Warisha Naseeb^1,^, Muhammad Kaleem Khosa^1,*^, Awal Noor ^2,^*, Sadaf Qayyum ^2^ and Shaowei Chen^3^

^1^Department of Chemistry, Government College University, Faisalabad, Pakistan

^2^Department of Basic Sciences, Preparatory Year Deanship, King Faisal University, Al-Hassa

31982, Saudi Arabia;

^3^Department of Chemistry and Biochemistry, University of California, 1156 High Street, Santa Cruz, CA 95064, United States

*Correspondence: [mkhosapk@yahoo.com](mailto:mkhosapk@yahoo.com) ( M.K.K.) [anoor@kfu.edu.sa](mailto:anoor@kfu.edu.sa) (A.N)

**Table S1.** Summary of elemental compositions (at. %) of the series of samples by XPS measurements.

| Sample | C | N | O | S | Ru | Co |
| --- | --- | --- | --- | --- | --- | --- |
| RuS_2_-CoO@SNG | 80.59 | 2.26 | 12.46 | 2.86 | 1.43 | 0.37 |
| RuS_2_@SNG | 78.90 | 2.06 | 12.06 | 6.04 | 0.89 | - |
| CoO@SNG | 90.64 | 1.24 | 5.02 | 2.96 | - | 0.13 |
| SNG | 91.96 | 0.43 | 4.33 | 1.72 | - | - |

**Table S2.** Binding energies (eV) and content (%) of Ru and Co series of sample by XPS.

| Sample | Ru (0) | Ru (4+) | Co (2+) |
| --- | --- | --- | --- |
| RuS_2_-CoO@SNG | 280.33 eV  0.72 at. %  461.91 eV  0.46 at% | 281.10 eV  0.84 at. %  463.52 eV  0.85 at % | 781.20 eV  0.204 at% |
| RuS_2_@SNG | 280.09 eV  1.09 at. %  461.42 eV  0.48 at% | 280.97 eV  0.09 at. %  462.90 eV  0.40 at% | - |
| CoO@SNG |  |  | 781.31 eV  0.08 at% |

**Table S3.** Binding energies (eV) and content (at. %) of S and N of sample by XPS.

| Sample | Sulfide | C-S-C | Pyridinic N | Pyrrolic N | Graphitic N |
| --- | --- | --- | --- | --- | --- |
| RuS_2_-CoO@SNG | 162.22 eV  0.43 at % | 163.82 eV  1.47 at % | 398.28 eV  0.65 at % | 399.68 eV  0.36 at % | 401.17 eV  0.33 at % |
| RuS_2_@SNG | 162.63 eV  2.86 at % | 164.51 eV  2.05 at % | 398.27 eV  0.78 at% | 399.86 eV  0.41 at% | 401.30 eV  0.86 at% |
| CoO@SNG | -- | 163.15 eV  2.71 at % | 397.93 eV  0.67 at% | -- | 400.53 eV  0.57 at% |
| SNG | -- | 163.59 eV  1.45 at % | 397.71 eV  0.17 at% | 398.27 eV  0.26 at% | 400.47 eV  1.02 at% |

**Fig. S1** HRTEM image of RuS_2_-CoO@SNG with the corresponding lattice fringes of RuS_2_ and CoO with live profile of the interplanar distance of RuS_2_ (200) lattice fringes and CoO(111) by Gatan DigitalMicrograph.

**Fig. S2** HRTEM image of RuS_2_@SNG with the corresponding lattice fringes of RuS_2_ with live profile of the interplanar distance of RuS_2_(200) lattice fringes by Gatan DigitalMicrograph.

**Fig. S3** (a) XRD pattern and (b) High-resolution XPS spectra of O 1s in RuS_2_-CoO@SNG, RuS_2_@SNG, CoO@SNG and SNG. Gray solid curves are experimental raw data and colored curves are deconvolution fits.

**Fig. S4** (a) Cyclic voltammograms of RuS_2_-CoO@SNG within the range of +0.1 to +0.2 V where no faradaic reaction occurred at difference scan rates in 1 M KOH (b) Variation of the double-layer charging currents at +0.15 V versus scan rate in 1 M KOH (c) Cyclic voltammograms RuS_2_-CoO@SNG within the range of +0.0 to +0.1 V where no faradaic reaction occurred at difference scan rates in 0.5 M H_2_SO_4_ (d) Variation of the double-layer charging currents at +0.15 V versus scan rate in 0.5 M H_2_SO_4_.

**Fig. S5** i-t curve of RuS_2_-CoO@SNG (a) at the applied potential of 153 mV in 1 M KOH (b) at potential of 95 mV in 0.5 M H_2_SO_4_. KSCN poisoning test of RuS_2_-CoO@SNG in (c) 1 M KOH (d) 0.5 M H_2_­SO_4_.
